# A response time model of the three-choice Mnemonic Similarity Task provides stable, mechanistically interpretable individual-difference measures

**DOI:** 10.3389/fnhum.2024.1379287

**Published:** 2024-08-29

**Authors:** Nidhi V. Banavar, Sharon M. Noh, Christopher N. Wahlheim, Brittany S. Cassidy, C. Brock Kirwan, Craig E. L. Stark, Aaron M. Bornstein

**Affiliations:** ^1^Department of Cognitive Sciences, University of California, Irvine, Irvine, CA, United States; ^2^Department of Political Science, University of California, Berkeley, Berkeley, CA, United States; ^3^Department of Psychology, University of North Carolina at Greensboro, Greensboro, CA, United States; ^4^Department of Psychology, Brigham Young University, Provo, UT, United States; ^5^Department of Neurobiology and Behavior, University of California, Irvine, Irvine, CA, United States; ^6^Center for the Neurobiology of Learning and Memory, University of California, Irvine, Irvine, CA, United States

**Keywords:** memory, pattern separation, Linear Ballistic Accumulator model, response times, fMRI

## Abstract

**Introduction:**

The Mnemonic Similarity Task (MST) is a widely used measure of individual tendency to discern small differences between remembered and presently presented stimuli. Significant work has established this measure as a reliable index of neurological and cognitive dysfunction and decline. However, questions remain about the neural and psychological mechanisms that support performance in the task.

**Methods:**

Here, we provide new insights into these questions by fitting seven previously-collected MST datasets (total *N* = 519), adapting a three-choice evidence accumulation model (the Linear Ballistic Accumulator). The model decomposes choices into automatic and deliberative components.

**Results:**

We show that these decomposed processes both contribute to the standard measure of behavior in this task, as well as capturing individual variation in this measure across the lifespan. We also exploit a delayed test/re-test manipulation in one of the experiments to show that model parameters exhibit improved stability, relative to the standard metric, across a 1 week delay. Finally, we apply the model to a resting-state fMRI dataset, finding that only the deliberative component corresponds to off-task co-activation in networks associated with long-term, episodic memory.

**Discussion:**

Taken together, these findings establish a novel mechanistic decomposition of MST behavior and help to constrain theories about the cognitive processes that support performance in the task.

## 1 Introduction

How do individuals encode objects in memory, and how does the distinctiveness of encoding affect behavioral expressions of recognition? These functions are thought to be supported by a process known as *pattern separation*, whereby similar sensory or latent inputs are projected into higher-dimensional space to create highly distinct patterns that support later discrimination among fine degrees of difference (Stark et al., 2019). Traditionally, this process has been attributed to the hippocampus, a critical brain structure for learning and memory (Marr, 1971; Long et al., 2016; Leal and Yassa, 2018; Stark et al., 2019), though more recent work suggests that the computation may be widely reflected across cortex (Amer and Davachi, 2023) and that mnemonic discrimination itself also depends on the whole brain (Nash et al., 2021; Wahlheim et al., 2022). Computational models predict that the more distinct object representations are (i.e., the “better” an individual is at pattern separating), the better an individual will be able to discriminate between objects that were seen previously and those that weren't. In particular, people who are better at pattern separating should be less susceptible to interference when novel stimuli are similar to previously seen stimuli. The ability to create distinct representations is of critical importance for behaviors that depend on episodic memory, such as context-sensitive decisions—if the decisions we make are guided by our previous experiences, we need a mechanism in place to be able to distinguish the appropriate, relevant experiences (Noh et al., 2023a,b).

The Mnemonic Similarity Task (MST) is a commonly used experimental task that aims to provide a behavioral index of an individual's tendency to pattern separate (Stark et al., 2019). The MST is a modified object-recognition task; in a popular variant it is often split into two distinct phases: study and test. During study, participants are given an incidental encoding task where they are presented with images of objects which they need to classify as belonging “indoors” or “outdoors.” Then, during test, participants are again presented with images, and their task is now to identify whether these images have been seen during the current experimental session. However, they are presented three different types of objects, each with the same frequency: *Repeats*, which are exactly the same objects they saw during study, *Lures*, which are objects similar to but not exactly the same as the study images, and *Foils*, which are objects that have never been seen before in the context of the experiment. There are multiple variants of the MST which differ in study design (two phase vs. continuous), number of responses (3: Repeat/Lure/Foil or 2: Repeat/Foil), and/or stimulus sets. In this paper, we consider only the “standard” three-alternative forced-choice (3AFC) two phase version of the MST. Subjects across experiments were not necessarily shown the same stimulus sets, but all stimulus sets were matched in their difficulty (Lacy et al., 2011).

The primary measure of memory discriminability used in the MST is the Lure Discrimination Index (LDI; Stark et al., 2019). Since the development of the MST, the LDI has been formalized in several ways. The most common formulation, and the one we employ in this study, is:


(1) $$\begin{array}{l} LDI=P\left(\text{Lure Response}|\text{Lure Trial}\right)-P\left(\text{Lure Response}|\text{Foil Trial}\right) \end{array}$$

Equation (1) can be thought of as the “hit rate” on lure trials corrected for the “bias” of incorrectly saying the foil is a lure (but correctly identifying the higher-order category of haven't-seen-before-in-the-experiment). This LDI has been shown to vary with age and across a wide range of clinical measures (Stark et al., 2019). However, while there is rich evidence for the external validity of the LDI insofar as correlating with activity in the hippocampal subfields ascribed to pattern separation (Stark et al., 2019), there has also been extensive debate about whether the LDI is “process pure,” as much as any cognitive parameter can be. Specifically, there is an open question as to whether LDI reflects pattern separation *per se* as opposed to distinguishing variation at both encoding and recollection (Liu et al., 2016). Indeed, it may be more precise to say that the LDI is a measure of mnemonic discrimination and its concomitant processes. Further, across the various parameterizations of the LDI, there is necessarily information loss. Any formulation of the LDI considers only one response type (out of three) and two trial types (out of three). If the key question of interest has to do with discrimination across various degrees of fidelity between old and new objects, it may make sense to use a measure that captures information about all three response types. In this paper, we propose a joint model of choice and response time that incorporates this full set of trials to estimate parameters linked to psychological mechanisms with distinct relationships to behavior. This model further allows us to decompose the LDI in order to begin to consider—through behavioral and neural data—what facets of mnemonic discrimination it is actually capturing.

## 2 Methods

### 2.1 Experiments and data

We analyze data from seven experiments collected by several researchers at different universities. We summarize them in the Table 1.

**Table 1:** MST experiments modeled.

**Experiment**	**Number of subjects**	**Number of trials**	**Source**
E1	*n* = 53	192	Stark-1
E2	*n* = 46	192	Stark-2
E3	*n* = 81	192	Stark-3
E4	*n* = 53	192	Stark-4
E5	*n* = 177	192	Noh-Lifespan
E6	*n* = 62	108	Wahlheim-Lifespan, RSFC
E7a	*n* = 47	192	Kirwan-Test-1
E7b	*n* = 47	192	Kirwan-Test-2

The *Source* column indicates the researchers who originally collected the data and any additional relevant information about the dataset. Experiment 5 contains a lifespan sample. Experiment 6 contains both a lifespan sample and resting state functional connectivity measures. Experiments 7a and 7b comprise of the same subjects who completed test twice—at least once after a one week delay (7b).

**Experiments 1 − 4: Stark et al**. The first set of experiments we analyze are from Dr. Craig Stark and colleagues (Stark et al., 2023). In this paper, Stark and colleagues contrasted several variants of the MST to assess the reliability and efficacy of measures. We consider a subset of these experiments that include the “full” or baseline version of the MST (number of test trials = 192, number of responses = 3). These experiments were collected across different individuals and did not necessarily use the same stimulus sets. However, all stimuli were matched for difficulty across each experiment. Here, we include analyses from four experiments in the paper.

**Experiments 5: Noh et al**. We analyze a subset of the data collected by Dr. Sharon Noh and colleagues (Noh et al., 2023a). This is again the baseline version of the MST but consists of a lifespan sample (age 18–84, number of test trials = 192, number of responses = 3), collected online via Amazon Mechanical Turk. We therefore also consider the relationship between the LDI, LBA parameters, and age.

**Experiments 6: Wahlheim et al**. We include data collected by Dr. Christopher Wahlheim and colleagues (Wahlheim et al., 2022). This is the baseline version of the MST but consists of fewer trials and a lifespan sample (age 18–80, number of test trials = 108, number of responses = 3). These data were also collected in participants who had, separately, undergone functional MRI during rest. Therefore, we also consider the relationship between the LDI, LBA parameters, age, and resting state functional connectivity. Functional neuroimaging data is analyzed on the basis of parcellated regions of interest including 37 regions that together comprise the default mode network, and 8 hippocampal subdivisions. The hippocampal ROIs were derived from the Schaefer parcellation of the MNI template (Schaefer et al., 2018), and the eight hippocampal regions were defined according to the Melbourne Subcortex Atlas (Tian et al., 2020, 2 mm group parcellation).

**Experiments 7a & 7b: Kirwan**. Finally, we analyze data provided by Dr. C. Brock Kirwan. This is the baseline version of the MST (number of test trials = 192), however, it was collected at two time points, the order of which was counterbalanced across conditions: all subjects completed a total of 2 study and 2 test segments. One half of subjects completed test immediately after study on session 1 (7a) followed by another study in the same session. They then took the test again one week after (7b). The other half of the subjects completed only study in session 1 and first completed test one week after (7a). They then completed another study and test (7b) in the same session. We examine the relative test-retest reliability of the LDI and LBA parameters.

### 2.2 Response time model: the Linear Ballistic Accumulator

We adapt the Linear Ballistic Accumulator (LBA; Brown and Heathcote, 2008) to model choices and response times in the MST. As with all sequential sampling models, the core process explained by the LBA is as follows. First, the stimulus is presented at the beginning of the trial. Then, after some initial processing e.g., identifying the trial image relative to its background, individuals start to accumulate evidence—sampling both the trial image and memory—until they have enough evidence to make a choice of either Repeat, Lure, or Foil. Finally, after they reach their internal decision, individuals may additionally take further time to execute their choice through motor movement. The time taken to do this, in addition to the initial processing, is called the “Non-Decision Time.” The LBA is a simple sequential sampling model that has the benefit of accommodating *n*-AFC experiments: we can fit *n* accumulators for *n* response types. It further assumes that evidence is independently and noiselessly accumulated for each response type. The LBA is a powerful accumulator that performs on par with more complicated sampling models that do not have the same assumptions (Brown and Heathcote, 2008). We further clarify that we use the LBA as a tool for measuring behavior that follows from representations that have ostensibly been pattern separated or not—not as an explicit model of pattern separation itself.

There are four main parameters of the LBA, all common to most models of sequential sampling: the drift rate *v*, or the rate of evidence accumulation/signal strength, the boundary *b*, or the amount of information needed for a response to be made, the non-decision time (NDT) τ, or the amount of time for perceptual and motor processing unrelated to decision deliberation, and the upper limit of the start point *A*—the bias toward making a particular response.

**Figure 1:**
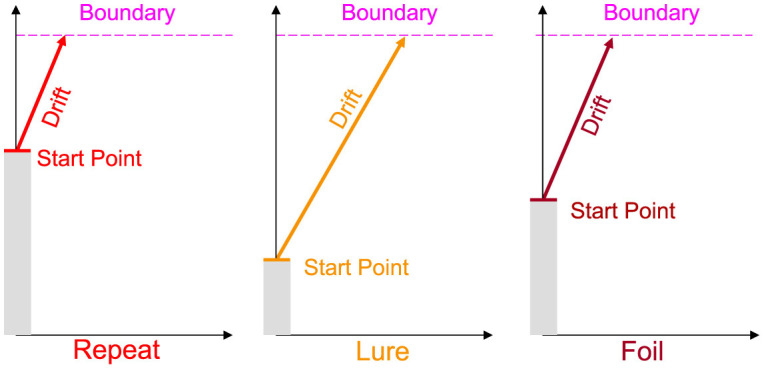
Schematic of the Linear Ballistic Accumulator. As the standard MST is 3AFC, we allow for three accumulators for each response type. We further allow the drift rate (rate of evidence accumulation) and the upper limit of the starting point (tendency to make a type of response) to vary for each subject and accumulator. Boundary (amount of evidence needed to make a response) and non-decision time (non-decision-relevant processes, not pictured) vary only at the subject level. Note this is a schematic and we do not impose any order constraints on drift rates or start points.

As shown in Figure 1, we allow the drift rate and start point to vary per subject *and* per accumulator, while allowing the boundary and NDT to only vary across subjects. As the three responses in the MST are distinct, it stands to reason that the evidence accumulated in favor of each response should be different. Similarly, the bias or predisposition to making one response over another should also intuitively vary as a function of response type. Otherwise, a fixed bias might suggest that subjects have uniform tendencies to respond Repeat, Lure, and Foil. As we discuss in the Results section, this indeed turns out not to be the case, with most subjects across experiments tending to respond Repeat disproportionately more often.

The LBA assumes that the drift rates are drawn from some Normal distribution and are sampled on each trial: Drift Rate~Normal(*v*_*i, r*_, *s*_*i, r*_) for subject *i* and response type *r*. In this paper, when we report values associated with the drift rate, we are talking specifically about the *mean* drift rate (*v*_*i, r*_). We fix the standard deviation of the drift rate (*s*_*i, r*_) to be 1 across all subjects and accumulators for model identifiability purposes.

The LBA assumes that starting points are uniformly distributed, sampled on each trial, and are numerically lower than the boundary: Start Point~Uniform[0, *A*_*i, r*_]*T*(0, *B*_*i*_) for subject *i* and response type *r*.

To further keep the model identifiable, we impose the same constraint on the mean of the drift rates and start point upper boundaries for each subject *i*: $\Sigma_{r=1}^{3}v_{i,r}=1$, $\Sigma_{r=1}^{3}A_{i,r}=1$. We fit a Bayesian implementation of the LBA in RStan (Stan Development Team, 2023). Stan uses a no U-turn sampling Hamiltonian Monte Carlo algorithm. In all our implementations, we run three chains for 7,000 iterations and allow for a burn-in of 3,500. The sum to one constraint is operationalized by allowing the drift rate mean and start point upper bound to be simplex types. We use the following relatively uninformative priors for the LBA parameters:


$$\begin{array}{r} \text{Boundary}\sim \text{Normal(0.5, 1) NDT}\sim \text{Normal(0.5, 1)}\\ \text{Drift Rate Mean}\sim \text{Normal(0.5,0.5)}\\ \text{Start Point Upper Bound}\sim \text{Normal(0.5,0.5)} \end{array}$$

## 3 Results

### 3.1 Raw response times and choices

To motivate the model-based analysis that is the main focus of our investigation, we first examined the pattern of response times and accuracy across stimulus types and experiments.

We see that across accuracy, median RT, response proportion, and LDI, experiments are comparable for all “standard” experiments where subjects completed test immediately after study (Tables 2–**5**). Summary statistics for subjects in Experiment 7b, which consists of a second test session, deviate. For example, in Table 2, we see that subjects performing this second test are most accurate at identifying foil stimuli as opposed to repeats and lures (*p* < 0.001).

**Table 2:** Subjects more accurately identify repeat and foil stimuli compared to lures across all standard experiments.

**Experiment**	**Accuracy: repeat**	**Accuracy: lure**	**Accuracy: foil**
E1	0.86 (0.18)	0.40 (0.32)	0.77 (0.30)
E2	0.87 (0.09)	0.41 (0.29)	0.78 (0.18)
E3	0.84 (0.15)	0.54 (0.23)	0.80 (0.13)
E4	0.84 (0.18)	0.44 (0.22)	0.82 (0.13)
E5	0.81 (0.14)	0.52 (0.24)	0.88 (0.13)
E6	0.92 (0.12)	0.31 (0.27)	0.86 (0.16)
E7a	0.87 (0.14)	0.64 (0.18)	0.87 (0.11)
E7b	0.34 (0.27)	0.29 (0.12)	0.71 (0.20)

We show median (IQR) accuracy for each response type. Across most experiments, subjects are more accurate when identifying repeat and foil stimuli vs. lure stimuli (Wilcoxon Rank Sign Test comparing repeat vs lure accuracy and foil vs. lure accuracy, E1−E7a: *p* < 0.001). This is not the case for experiment 7b, delayed test, where subjects were most accurate at identifying foil stimuli (though see **Table 4**: they were also most often making Foil responses).

**Figure 2:**
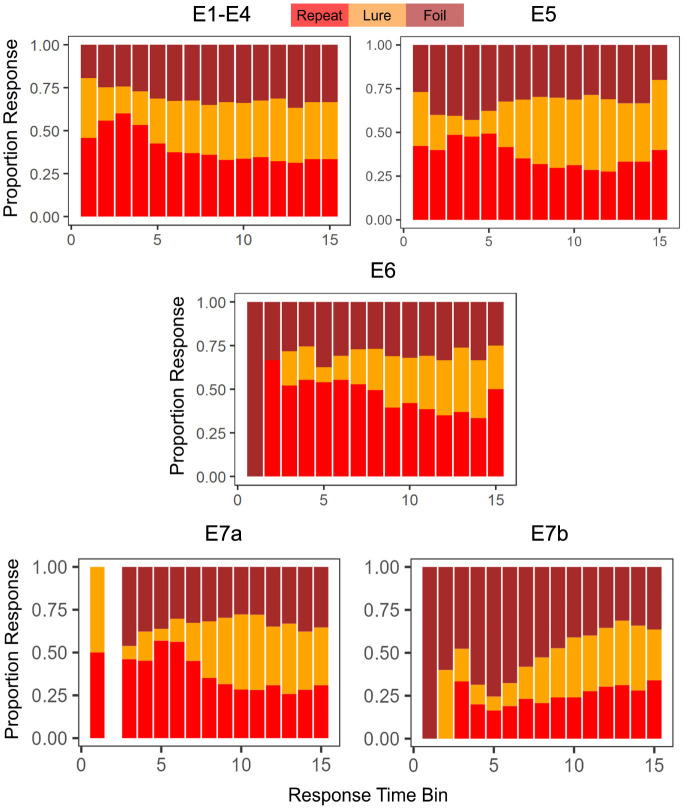
Choice proportions change as a function of response time. We find, in general, that faster choices tend to be *Repeats*. The empty bar in E7a indicates that no responses were made with a RT that fit in the time bin.

Another point of interest concerns the evolution of choice and response time over the course of the experiment. Previous research on memory and response time suggests that choices that are easier (more accessible in memory) should be faster, and choices that are more difficult should take longer (Collins and Loftus, 1975). In the MST, the repeated stimuli are typically considered the easiest to identify (after all these are the stimuli that have already been seen before in the context of the experiment). Indeed, as we demonstrate in Figure 2, this appears to be the case, though there is variability across experiments. When we collapse across Experiments 1 − 4, with the exception of the very fastest responses, we find that subjects tended to label a stimulus as a Repeat most often when making a quick decision (*RT* < 1.38*s*, the median RT). For slower decisions (*RT*>1.38*s*), the highest frequency response was Foil. In Experiment 5, we find that decisions faster than the median RT of 1.19*s* were similarly majority Repeat, but that slower responses were most often Lures. As shown in Table 3, the median RTs for Repeat and Foil responses are lower than the median RT for Lure responses in most experiments, explaining why we see a majority of slow Lure responses despite Table 4 showing a low overall proportion of Lure responses. In contrast, in Experiment 6, we found that the majority of responses over time were Repeats (with the exception of 4 later RT bins out of 15 (*RT*>1.29*s*) which had a majority response of Foil), In Experiment 7a, we found that responses faster than the median were, like Experiments 1–6, Repeats and that the majority of slow responses were Lure. Interestingly, in Experiment 7b, with the exception of the 13th and 14th RT bins where the most common response was Lure, the overwhelmingly common response was Foil.

**Table 3:** Subjects are faster when making Repeat and Foil responses compared to Lure across all standard experiments.

**Experiment**	**Med RT: repeat**	**Med RT: lure**	**Med RT: foil**
E1	1.17 (0.28)	1.34 (0.32)	1.31 (0.28)
E2	1.21 (0.22)	1.34 (0.24)	1.26 (0.23)
E3	1.25 (0.23)	1.38 (0.29)	1.35 (0.31)
E4	1.72 (0.36)	1.99 (0.42)	1.80 (0.36)
E5	1.11 (0.20)	1.37 (0.31)	1.13 (0.22)
E6	1.05 (0.21)	1.30 (0.34)	1.11 (0.26)
E7a	1.09 (0.16)	1.29 (0.18)	1.17 (0.17)
E7b	1.34 (0.20)	1.37 (0.14)	1.15 (0.20)

We show median (IQR) RT for each response type. We find that, on average, participants tend to take longer when they make a Lure response, especially when compared to when they make a Repeat response (Wilcoxon Rank Sign Test comparing median RT for repeat vs. lure responses and foil vs. lure responses for experiments 1 − 7a, *p* < 0.001 except for E1,2,3 Lure vs. Foil which were not statistically significant).

**Table 4:** Subjects most often make Repeat responses across all experiments.

**Experiment**	**Prop: repeat**	**Prop: lure**	**Prop: foil**
E1	0.44 (0.19)	0.26 (0.13)	0.30 (0.09)
E2	0.47 (0.12)	0.23 (0.12)	0.30 (0.06)
E3	0.41 (0.12)	0.28 (0.12)	0.31 (0.06)
E4	0.43 (0.11)	0.24 (0.09)	0.33 (0.05)
E5	0.40 (0.11)	0.25 (0.12)	0.34 (0.06)
E6	0.51 (0.11)	0.19 (0.13)	0.32 (0.06)
E7a	0.39 (0.08)	0.28 (0.08)	0.31 (0.05)
E7b	0.22 (0.17)	0.25 (0.09)	0.51 (0.19)

We show median (IQR) choice proportions for each response type. We see that in Experiments 1–7a, participants to most often classify stimuli as repeat (Wilcoxon Rank Sign Test comparing response proportions for repeat vs. lure responses and repeat vs. foil responses, *p* < 0.001 for all experiments). Recall that the true proportion of repeated stimuli presented during test is 0.33. Interstingly, in Experiment 7b, subjects most often classify stimuli as Foil (*p* < 0.001).

### 3.2 Model fits

All results reported are from models that pass all metrics of convergence: *Rhat* < 1.01, chains converge in traceplots, and no autocorrelation.

#### 3.2.1 Posterior summaries

**Figure 3:**
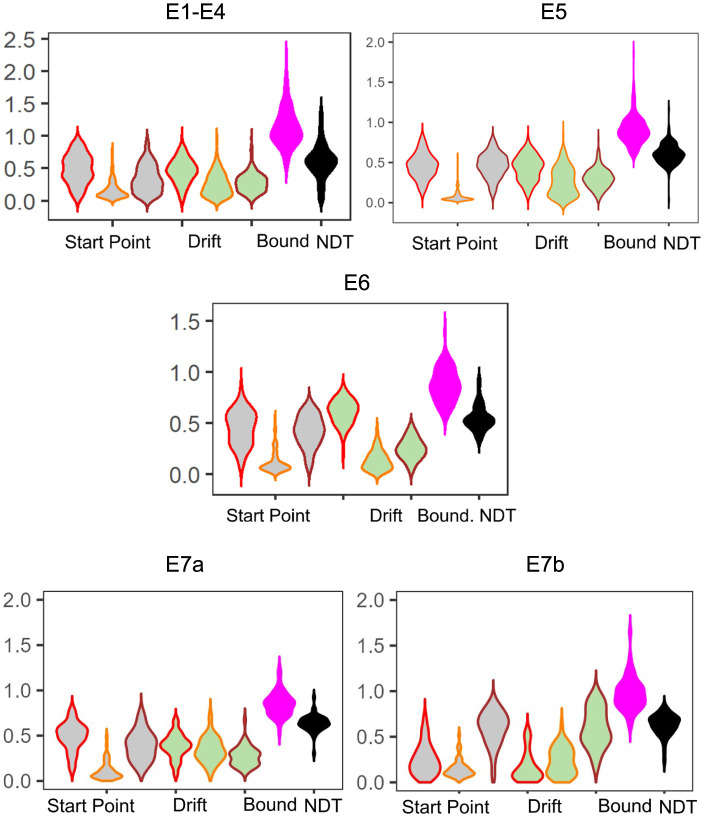
Model posteriors for all experiments. We find overall that LBA posteriors follow qualitatively the same patterns across experiments. The left most three gray violins are the start point upper bound (color coded by response type: Repeat, Lure, and Foil). Next are the drift rates, similarly color coded. Finally, we have the boundary and non-decision time.

We find that the LBA parameters across the experiments show generally the same patterns: start point upper limits and drift rates for Lure responses tend to be the lowest (median SP E1–4: 0.12, E5: 0.05, E6: 0.08 and median drift E1–4: 0.23, E5: 0.24, E6: 0.12), whereas start point upper limits and drift rates for Repeat responses (median SP E1–4: 0.49, E6: 0.47, E6: 0.47 and median drift E1–4: 0.47, E5: 0.45, E6: 0.63), tend to be comparable with Foil responses (median SP E1–4: 0.30, E5: 0.46, E6: 0.43 and median drift E1–4: 0.27, E5: 0.30, E6: 0.25; Figure 3). This is consistent with our expectations given the patterns observable in the response times. We also believe that this suggests reasonable recovery of information by our model: while there can be great heterogeneity across individuals, with the exception of E5 and E6, which contain a subset of older adults, there is no reason to expect qualitative differences in model fits across experiments. After all, the test phase of the experiment has the same structure across all the datasets we consider, with the exception of E6 which has fewer trials. This consistency is particularly of interest given how much variability there is in the LDI across experiments (see Table 5), suggesting that the parameters estimated using the LBA may be more stable individual-difference measures. We now turn to this question more directly.

**Table 5:** Lure discrimination indices for each experiment.

**Experiment**	**Lure discrimination index**
**E1**	**0.18 (0.40)**
E2	0.21 (0.33)
E3	0.34 (0.28)
E4	0.28 (0.25)
E5	0.36 (0.30)
E6	0.17 (0.30)
E7a	0.50 (0.23)
E7b	0.08 (0.12)

We show median (IQR) LDI, the current standard metric for summarizing choice behavior in the MST. We find no statistical differences in median LDI across experiments, excepting 7b, which was performed at a delay.

#### 3.2.2 Relating the LDI to LBA parameters

**Figure 4:**
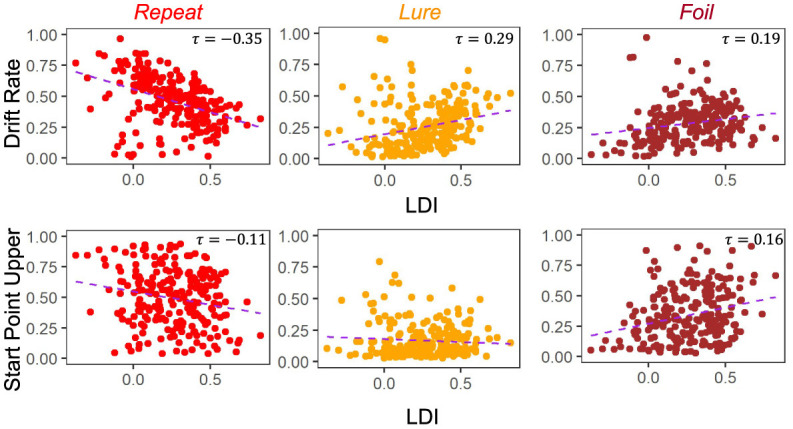
LDI correlates with drift rate *and* start point upper bounds in E1–E4. We collapse across all four experiments (*n* = 233) and correlate mean drift rate and start point upper bound with LDI. Kendall tau correlations shown in plots are statistically significant (all *p* < 0.001 except Start Point Repeat-LDI which has *p* = 0.01) after adjusting for multiple comparisons. We find statistically significant correlations between the LDI and drift rates for all accumulators. We also find significant correlations between the LDI and Repeat and Foil accumulator start point upper bounds.

A key goal of this work is to try to compare the relationships between the LDI and our model parameters. Of particular interest is the relationship between LDI and drift rate, and LDI and start point upper bound. This relates to the question briefly considered in the introduction: is the LDI capturing a signal of recognition memory? How much of it is conflated with other processes? If the LDI correlates *only* with the drift rate, which is the LBA's measure of signal strength, it suggests that the LDI may indeed be largely a measure of how distinct people's internal representations are. The more distinct the internal representation, the stronger the internal signal during evidence accumulation. Conversely, if the LDI correlates *only* with the start point upper bound, which is the LBA's measure of a tendency to make a particular response, it suggests that the LDI may be largely capturing something else, such as an individual or ephemeral tendency to respond one way rather than the others. However, it is rare for any one cognitive process to work in isolation, and indeed we find that the LDI correlates with *both* the accumulator drift rates and the accumulator starting point upper bounds in Experiments 1 − 4 (Figure 4). We collapse data across all four experiments as they were not designed to be meaningfully different across from each other. This results in a total sample size of 233 subjects.

Across experiments, we found strong statistically significant correlations between the LDI and drift rates for each accumulator [Kendall's tau all: corr(LDI, Repeat drift rate) = −0.35 (*p* < 0.001), corr(LDI, Lure drift rate) = 0.29 (*p* < 0.001), corr(LDI, Foil drift rate) = 0.19 (*p* < 0.001)]. Similarly, we found statistically significant correlations between the LDI and start point upper bounds for the Repeat and Foil accumulators [Kendall's tau: corr(LDI, Repeat start point upper bound) = −0.11 (*p* = 0.01), corr(LDI, Foil start point upper bound) = 0.16 (*p* < 0.001)]. We speculate that the lack of significant relationship between the LDI and start point upper bounds for Lure accumulators may be driven by the fact that there are overwhelmingly fewer lure responses compared to the other response types across all experiments. We corrected for multiple comparisons using the Bonferroni-Holm procedure. We note that the sign differences in the correlations between Repeat vs. Lure and Foil are as expected because the LDI, as defined in this paper, is parametrized to only capture signal from Lure and Foil stimuli. The LDI is explicitly calculating a “signal” of how well an individual discriminates between items that haven't in their totality been seen before in the context of the experiment. This is the complementary process to recognizing old items. Finally, we find a positive correlation between LDI and Non-Decision Time (τ = 0.17, *p* < 0.001): the better the memory discrimination, the longer the non-decision relevant processing.

**Figure 5:**
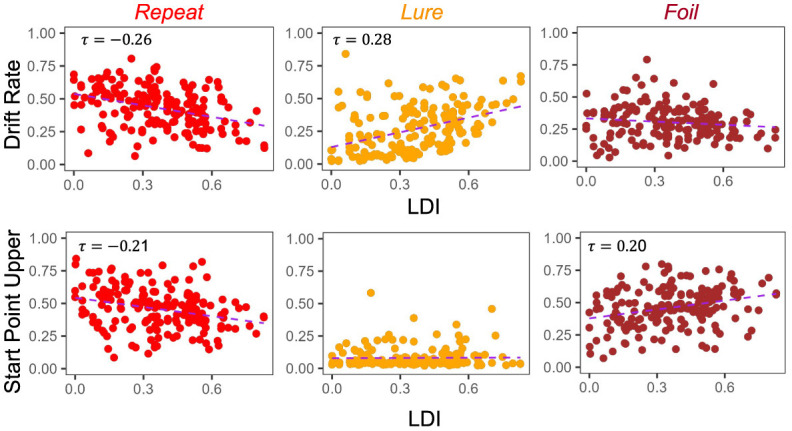
E5: LDI correlates with drift rate *and* start point upper bounds in lifespan sample 1. In the first dataset comprised of older and younger adults, we find similar qualitative relationships between the LDI and accumulator drift rates/start point upper bounds. Kendall tau correlations shown in plots are statistically significant (all *p* < 0.001) after adjusting for multiple comparisons.

**Figure 6:**
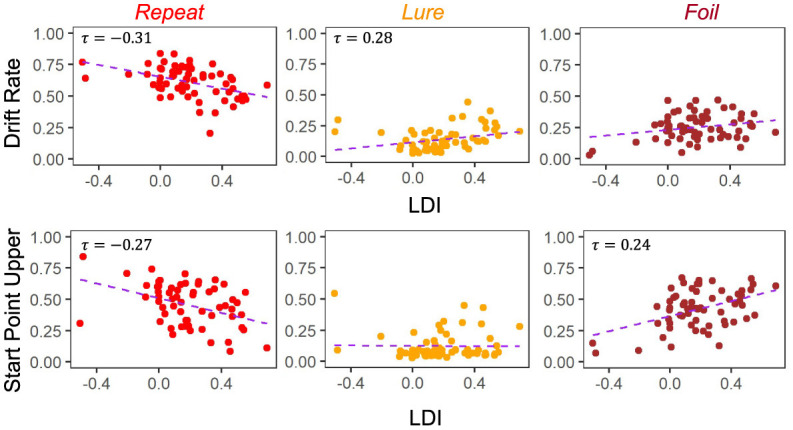
E6: LDI correlates with drift rate *and* start point upper bounds in lifespan sample 2. In the second, unrelated, dataset comprised of older and younger adults, we find similar qualitative relationships between the LDI and accumulator drift rates/start point upper bounds. Kendall tau correlations shown in plots are statistically significant (all *p* < 0.001) after adjusting for multiple comparisons.

The degree to which these correlational relationships hold across experiments is, however, variable. In particular, in the datasets with older adult participants—E5 (Figure 5) and E6 (Figure 6)—we find that the Repeat and Lure accumulator drift rates correlate significantly in the same way as in Figure 4 [Kendall's τ: E5: corr(LDI, Repeat drift rate) = −0.26, *p* < 0.001 and E6: corr(LDI, Repeat drift rate) = −0.31, *p* < 0.001; E5: corr(LDI, Lure drift rate) = 0.28, *p* < 0.001 and E6: corr(LDI, Lure drift rate) = 0.28, *p* = 0.001], but that there is no significant linear relationship between the LDI and Foil accumulator drift rate (Figures 5, 6). Conversely, we find exactly the same statistical patterns in the relationship between LDI and the starting point upper bounds [Kendall's tau: E5: corr(LDI, Repeat start point upper bound) = −0.21, *p* < 0.001 and E6: corr(LDI, Repeat start point upper bound) = −0.27, *p* = 0.002; E5: corr(LDI, Foil start point upper bound) = 0.20, *p* < 0.001 and E6: corr(LDI, Foil start point upper bound) = 0.24, *p* = 0.005]. We also find no relationship between NDT and LDI (E5: τ = 0.03, *p* = 0.59 and E6: τ = −0.04, *p* = 0.64) and likewise between Boundary and LDI (E5: τ = −0.13, *p* = 0.009 not significant after multiple comparison correction and E6: τ = −0.04, *p* = 0.64). We explicitly consider LDI-LBA correlations for Experiments 7a and 7b later when examining external validity.

##### 3.2.2.1 Comparing correlation strengths

To formally compare correlation strengths, we resampled the data and calculate Kendall's τs and the differences between each pair of τs (e.g., τ_*A*_−τ_*b*_). In particular, we wanted to test whether the correlations between the LDI and Drift Rates were stronger than the correlations between the LDI and Start Points. We then examined whether the 95% confidence interval distributions of the differences between each pair of correlations included zero. If they did not include zero, we interpreted this as evidence for rejection of the null (no difference between the correlations).

**Figure 7:**
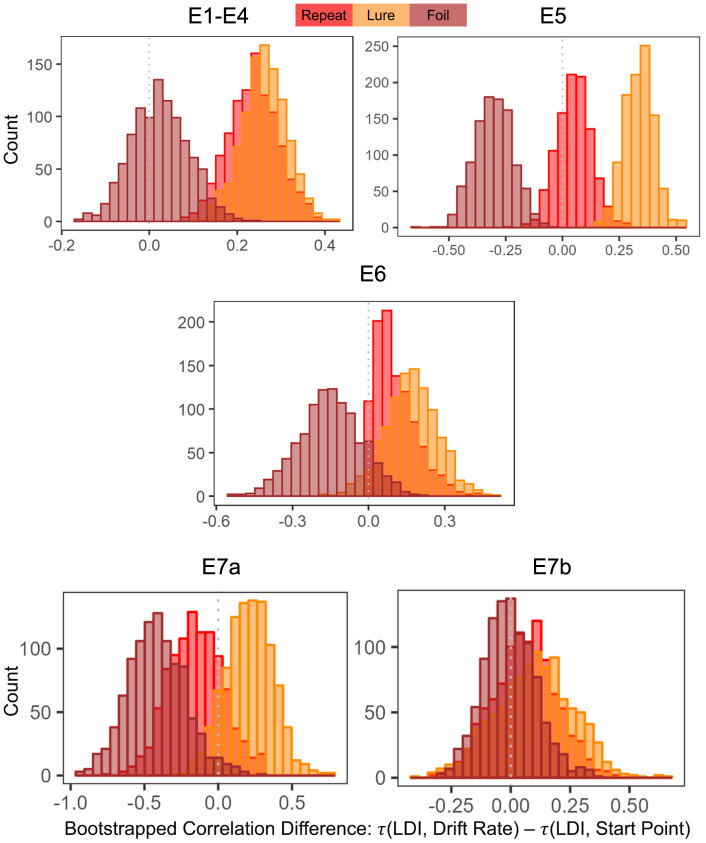
Histograms of bootstrapped correlation differences between LDI and drift rate, and LDI and start point. E1–E4: LDI-Drift rate correlations for Repeat and Lure accumulators are stronger than LDI-Start Point correlations. In E5, we find that LDI-Drift Rate for Lure and Foil accumulators are stronger than LDI-Start Points. E6, 7a, 7b show no significant correlation differences.

For Experiments 1–4, we found that the 95% CIs for the correlation difference between LDI-Drift Rate and LDI-Start Point for the Repeat and Lure accumulators did not contain 0 [Repeat accumulator correlation difference (0.13, 0.35), Lure accumulator correlation difference (0.16, 0.36), Figure 7]. However, this was not the case for the Foil accumulator [Foil accumulator correlation difference (−0.10, 0.15)]. We therefore reject the null hypothesis that the correlations between the LDI-Drift Rate and LDI-Start Point for the Repeat and Lure accumulator are the same. For Experiment 5, we find that the 95% CIs for the LDI-Drift Rate and LDI-Start Point correlation differences for Lure and Foil did not contain 0 [Lure: (0.20, 0.46), Foil: (−0.47, −0.13)]. This corresponds to the correlations shown in Figure 5—we find significant correlation with LDI for Lure responses only with drift rate, and with Foil responses only with start point. There we reject the null hypothesis equating correlation strengths for Lure and Foil responses but not for Repeat [95% CI (−0.21, 0.11)]. For Experiment 6, however, we found that none of the 95% CIs excluded zero [Repeat accumulator correlation difference (−0.29, 0.21), Lure accumulator correlation difference (−0.05, 0.37), Foil accumulator correlation difference (−0.39, 0.09)]. Formally, this suggests that we cannot reject the null of no difference in correlation strength between LDI and respective accumulator drift rate/start point upper boundary, however, we point out that for the Lure and Repeat accumulators, the 95% CIs only just include 0. Likewise, all constructed intervals include 0 for Experiments 7a and 7b, as is evident in Figure 7.

Overall, this analysis suggests that the LDI may indeed be more of a measure of signal strength than response bias, consistent with recent work in the two-choice version of this task (Chwiesko et al., 2023), though with considerable variability in this relationship across populations.

### 3.3 Age, the LDI, and LBA parameters

Another way to consider how valuable our model is is to consider measures outside the LDI. Experiments 5 and 6 are both lifespan samples, containing subjects as young as 18 and as old as 84. Given the typically demonstrated relationship between age and LDI (and mnemonic discrimination more broadly) (Stark et al., 2019; Foster and Giovanello, 2020; Koen et al., 2020), we considered whether the relationship between the LDI and LBA parameters was modulated by age.

For both experiments, we dichotomize adults into one of two age groups: younger or older (as in Wahlheim et al., 2022). These groups are comparable across both experiments: *younger* E5 median age 23 (*IQR* = 12) and *n* = 98; E6 median age 21 (*IQR* = 3) and *n* = 34 and *older* E5 median age 62 (*IQR* = 7) and *n* = 79; E6 median age 69.5 (*IQR* = 8.5) and *n* = 28. We split age into two groups due to bimodality in the spread of the ages in both experiments.

Younger adults had a median accuracy of E5: 0.73 (*IQR* = 0.10) and E6 0.69 (*IQR* = 0.11) compared to older adults E5: 0.69 (*IQR* = 0.09) and E6: 0.66 (*IQR* = 0.08). Younger adults were also faster than older adults [E5: median RT young = 1.11 (0.15) vs. median RT old = 1.27 (0.16); *W* = 6,125.5, *p* < 0.001 and E6: median RT young = 1.03 (0.16) vs. median RT old = 1.19 (0.16); *W* = 779.5, *p* < 0.001—we report the results of a Wilcoxon Rank Sum Test due to non-normality in the distribution of untransformed RTs.].

We find significant differences in lure discrimination as a function of age group, as Wahlheim et al. (2022) also find in their original analysis of E6. Younger adults have a significantly higher LDI than older adults [E5: median LDI young = 0.41 (0.26) vs. median LDI old = 0.32 (0.28); *W* = 3,003.5, *p* = 0.01 and E6: median LDI young = 0.27 (0.26) vs. median LDI old = 0.09 (0.18); *W* = 693.5, *p* < 0.001].

#### 3.3.1 Differences in LBA parameters

**Figure 8:**
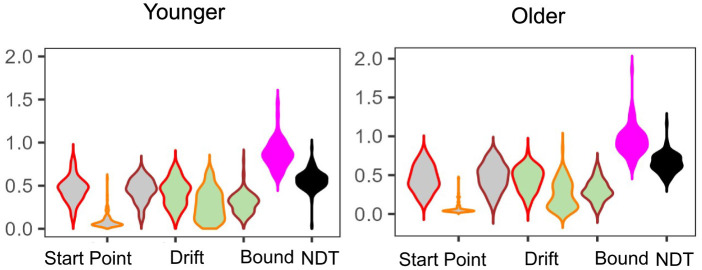
E5: Posterior model fits as a function of age group. Overall, parameter estimates are largely comparable across the two age groups. We only find significant group differences in the Non-Decision Time parameter.

In E5 (Figure 8), and E6, we only find statistically significant differences in non-decision time, with older adults taking longer with non-decision-related processes than younger adults [E5: median NDT young = 0.56 (0.12) vs. median NDT old = 0.65 (0.16); *W* = 5,525 and E6: median NDT young = 0.49 (0.08) vs. median NDT old = 0.60 (0.16); *W* = 794, both *p* < 0.01]. As we show in our earlier analysis, the LDI is meaningfully correlated with both drift rates and start point upper boundaries. Therefore, while the LDI is different as a function of age group, it is not necessarily surprising that its relationship with the relevant LBA parameters do not differ across age groups: they are putative *components* of the LDI. We further assessed how age directly correlated with LBA parameters. We found, in E5, that age differentially correlates with lure drift rate (younger τ = 0.21 and older τ = −0.22, *p* < 0.001) and, only in the younger group, also correlates with repeat drift rate (τ = −0.26, *p* < 0.01) and NDT (τ = 0.28, *p* < 0.001).

Interestingly, when we compared correlations between the LDI and LBA parameters as a function of age group, only a handful of statistically significant correlations remain after adjusting for multiple comparisons, and only in E5 (Tables 6, 7). In younger adults, the Repeat and Lure drift rates correlate significantly with LDI in the same manner as Experiments 1 − 4 whereas in older adults, the Repeat and Foil start points correlate significantly with LDI. However we note that several correlations are marginally significant, in particular the LDI and Repeat accumulator drift rate in older adults. Finally, we did not find any meaningful differences in the correlations between age groups (e.g., the LDI-Drift Repeat correlation for younger adults was not significantly different from the LDI-Drift Repeat correlation for older adults; this was the case for all parameter correlations).

**Table 6:** Younger adult LDI-LBA correlations follow the same qualitative patterns as in E1–E4 for drift rate and start point.

**Parameters correlated**	**E5**	***p*** **-value**	**E6**	***p*** **-value**
	**Correlation**		**Correlation**	
LDI-drift *Repeat*	−0.35	< 0.001	−0.26	0.03
LDI-drift *Lure*	0.41	< 0.001	0.28	0.02
LDI-drift *Foil*	−0.14	0.04	0.07	0.57
LDI-start point *Repeat*	−0.15	0.03	−0.18	0.14
LDI-start point *Lure*	−0.02	0.76	−0.04	0.72
LDI-start point *Foil*	0.15	0.03	0.13	0.27
LDI-boundary	−0.18	0.01	−0.02	0.86
LDI-NDT	0.19	0.01	0.05	0.68

The LDI-Drift Repeat and LDI-Drift Lure correlations are statistically significant after multiple comparison corrections only in E5, which has 64 more subjects than E6. The remaining Kendall τ correlations are not statistically significant, however, after the correction.

**Table 7:** Older adult LDI-LBA correlations also follow similar qualitative patterns as in E1–E4.

**Parameters correlated**	**E5**	***p*** **-value**	**E6**	***p*** **-value**
	**Correlation**		**Correlation**	
LDI-drift *Repeat*	−0.14	0.08	−0.36	0.01
LDI-drift *Lure*	0.20	0.01	0.16	0.24
LDI-drift *Foil*	−0.04	0.59	0.20	0.14
LDI-start point *Repeat*	−0.32	< 0.001	−0.22	0.10
LDI-start point *Lure*	−0.07	0.37	0.11	0.40
LDI-start point *Foil*	0.30	< 0.001	0.20	0.13
LDI-boundary	−0.02	0.76	−0.06	0.68
LDI-NDT	−0.06	0.46	0.34	0.01

The only significant correlations are again in E5 but, interestingly, between LDI and start point for Repeat and Foil responses. None of the remaining correlations are statistically significant, however, after adjusting for multiple comparisons.

### 3.4 Test-retest and external validity

#### 3.4.1 Test immediately and test after delay

To first consider the consistency of the LDI and LBA parameters, we compare model fits between Experiments 7a and 7b. The same subjects completed the standard MST twice where the second test session took place one week after the first study session. Our key question of interest lies in how our choice-only and choice-and-rt parameters vary over these two sessions. First, we note that in both experiments, we do *not* see the same qualitative relationships between LDI and the LBA parameters (e.g., negative correlation with Repeat responses and positive for Lure/Foil). Interestingly, the LDI does not significantly correlate with any LBA parameters in either the immediate test (E7a) or the delayed test (E7b). However, as our primary interest in these data lies in the difference in parameter values, we do not believe this lack of correlation to be a principal problem.

We then calculated how well the parameters rank-correlated within subject. We found no significant correlation between the LDIs across both experiments (τ = 0.09, *p* = 0.39). However, we found several significant correlations within LBA parameters including start point for the Repeat and Foil accumulators, non-decision time, and boundary (start point repeat accumulator τ = 0.34, start point foil accumulator: τ = 0.30, NDT: τ = 0.36; Boundary: τ = 0.41 all *p* < 0.001). We note that none of the significant correlations include any of the parameters that measure signal strength. Systems consolidation theory suggests that mnemonic representations transfer to long-term storage across the delay. Thus we may expect subjects to rely more on “gisty” memory for weakly-encoded items, while more strongly encoded items may undergo sharpening, consistent with our interpretation of start point.

**Figure 9:**
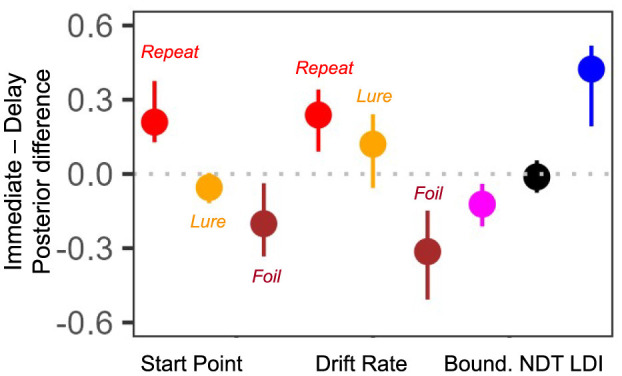
E7a,b: Posterior differences between immediate test and delayed test. We plot median (IQR) differences and find that the Lure accumulator start point and NDT have the smallest magnitude changes across the two experimental sessions.

Finally, we compared differences in the posterior means for each subject for all parameters of interest. That is, we subtracted the posterior value inferred during the second test from the posterior value inferred during the first test. Recall that the previous paragraph considered *rank* correlations while here we consider the magnitude of the parameter. The more variable a parameter, the greater the magnitude of the summary level difference. As we find in Figure 9, the start point for Lure responses and the NDT have the smallest magnitude of difference [median difference(IQR) start point Lure: −0.05 (0.12) and NDT: −0.01 (0.13)]. Interestingly, the drift rate for the Foil accumulator and the LDI have the largest magnitude of difference [median difference(IQR) drift rate Foil: −0.31 (0.36) and LDI: 0.42 (0.33)]. This seems consistent with our model free analyses earlier: in Tables 2, 3 we saw that subjects were most accurate and fastest when making Foil responses in E7b and that there was a large difference in median LDI between E7a and E7b (Table 5). This analysis provides initial evidence suggesting that parameters inferred via the LBA may be more stable than using just the LDI.

#### 3.4.2 Hippocampal resting state functional connectivity

We finally consider the more explicit question of external validity. As the LDI has been shown to be connected to not only hippocampal activity (Stark et al., 2019) but also more broadly neural activity across the brain (Wahlheim et al., 2022), we explore how our model-derived parameters correlate with these regions. In particular we compare and contrast how the LDI relates to neural activity with our LBA parameters. In their original paper, Wahlheim and colleagues demonstrated that resting state functional connectivity (RSFC) in the Default Mode Network predicts LDI (Wahlheim et al., 2022). We use exactly the same regions and parcellations as (Wahlheim et al., 2022). Our analyses are exploratory, constrained by the data from the initial paper, and uncorrected. Here, we first examine the relationship between resting state functional connectivity in the hippocampus (eight regions: left/right medial head, left/right lateral head, left/right body, and left/right tail) and the LDI/LBA parameters. We correlate LDI and LBA parameters with a matrix of Fisher's *z*-transformed correlation coefficients of hippocampal connectivity [procured from OSF and preprocessed by Wahlheim et al. (2022)].

As the MST is designed to capture a process most often attributed to the hippocampus, and we see a behavioral relationship between the LDI and some LBA parameters, we first considered if the LDI and LBA parameters correlated with hippocampal RSFC.

Recall from the previous section that in this dataset (not accounting for the differently aged subgroups), we found that LDI correlated with both mean drift rate (Repeat, Lure) and start point upper bound (Repeat, Foil). We therefore wanted to examine whether similar patterns held with resting state functional connectivity: how does LDI correlate with hippocampal RSFC, how do LBA parameters correlate with hippocampal RSFC, and what is the intersection and nodes of divergence between the two?

**Figure 10:**
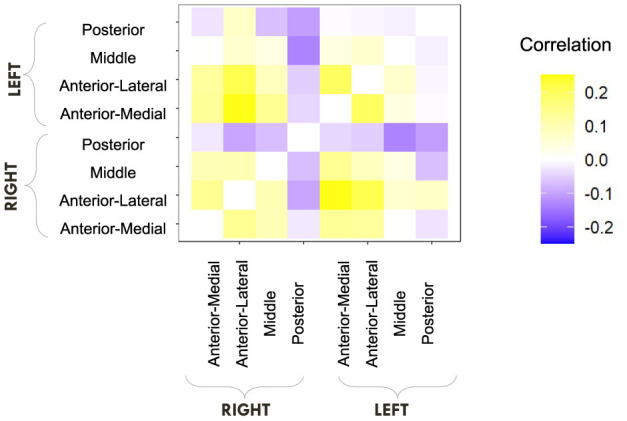
E6: LDI tends to correlate negatively with posterior hippocampal RSFC and positively with other regions. We show a symmetric correlation matrix plot where each square represents the correlation between the row-column hippocampal resting state functional connectivity and the LDI. Cells colored darker yellow show stronger positive correlations, and darker purple stronger negative correlations.

**Figure 11:**
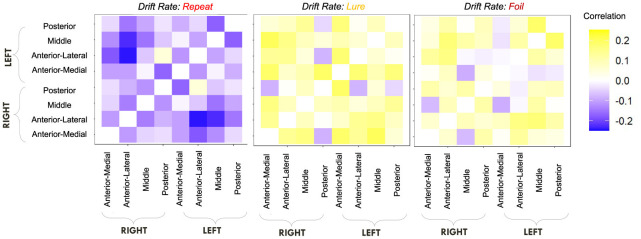
E6: Lure accumulator drift rates mostly correlate positively with hippocampal RSFC while Repeat accumulator drift rates correlate negatively. We show a symmetric correlation matrix plot where each square represents the correlation between the row-column hippocampal resting state functional connectivity and the respective accumulator drift rate.

**Figure 12:**
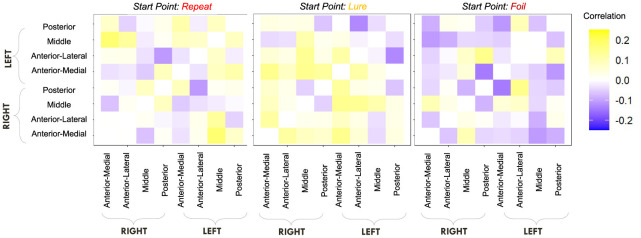
E6: Start point—hippocampal RSFC correlations. We show a symmetric correlation matrix plot where each square represents the correlation between the row-column hippocampal resting state functional connectivity and the respective accumulator drift rate. We also see greater variability in the qualitative patterns in the start point—RSFC correlations (i.e., evidence of both negative and positive correlations), compared to drift rate—hippocampal RSFC.

We first computed raw Pearson correlations between hippocampal RSFC and our behavioral parameters of interest. In Figure 10, we find that the LDI correlates negatively with posterior hippocampal RSFC and positively with other, more anterior, regions, perhaps in line with the empirically observed representational specificity gradient within the hippocampus. In Figure 11, we show that Repeat and Lure accumulator drift rates differentially correlate with hippocampal RSFC: with Repeat drifts correlating negatively and Lure drifts correlating mostly positively. Finally, in Figure 12, we find less clear directional patterns between accumulator start points and hippocampal RSFC. Further, absolute values of the correlations suggest that start point—RSFC correlations may be weaker than drift rate—RSFC.

We find that the LDI, drift rates for all accumulators, upper boundary for the Repeat accumulator start point, and non-decision time all show uncorrected thresholded (*p* < 0.05) Kendall τ correlations with various RSFC regions in the hippocampus (Table 8). Of particular interest is that *only* the drift rate for the Repeat and Lure accumulator correlated with the same RSFC regions as the LDI (and in directions consistent with previous independent datasets, Experiments 1 − 4: LDI negatively correlated with Repeat accumulator and positively correlated with Lure). We further highlight that each parameter correlated with hippocampal RSFC correlates *in the same direction*—for example, Repeat accumulator drift rates are always negatively correlated with RSFC but Lure accumulator drift rates are all positively correlated. This suggests that the correlations we recover are not necessarily spurious (in which case we may expect variability in the directionality of the correlations—contrast for example, Figure 11 vs. Figure 12). The thresholded analysis, then, could further support our boostrapped correlation analysis which suggested that LDI be relatively more a measure of signal strength compared to response bias.

**Table 8:** Thresholded hippocampus resting state functional connectivity and LDI/LBA parameters: *Only drift rates correlate with same RSFC regions as LDI*.

**Parameter**	**Regions**	**Correlation**
**LDI**	**Anterior lateral LH - Anterior lateral RH**	**τ = 0.21**

	Anterior medial LH - Anterior lateral LH	τ = 0.20
Drift: Repeat	Anterior lateral LH - Anterior lateral RH	τ = −0.24
	Anterior lateral RH - Middle LH	τ = −0.23
	Middle LH - Posterior LH	τ = −0.17
Drift: Lure	Anterior medial LH - Anterior lateral LH	τ = 0.18
	Anterior medial RH - Middle LH	τ = 0.20
Drift: Foil	Anterior lateral RH - Middle LH	τ = 0.17
	Middle LH - Posterior LH	τ = 0.18
Start point: Repeat	Anterior medial RH - Middle LH	τ = 0.18
Start point: Lure	-	-
Start point: Foil	-	-
Boundary	-	-
Non-decision time	Anterior medial LH - Anterior lateral LH	τ = −0.19
	Anterior lateral LH - Anterior lateral RH	τ = −0.21

Each row delineates which parameter correlated significantly (*p* < 0.05) with RSFC between the two listed hippocampus subregions. LH and RH are shortform for Left Hemisphere and Right Hemisphere, respectively.

##### 3.4.2.1 Default mode network resting state functional connectivity

**Figure 13:**
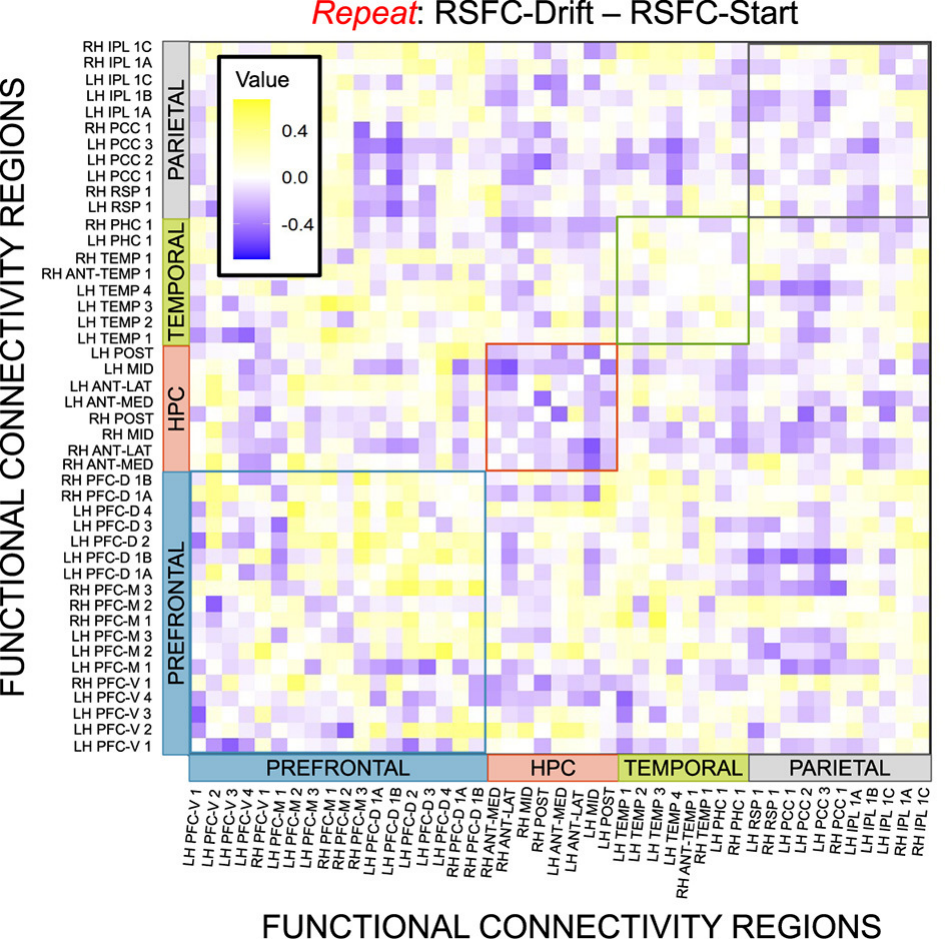
E6: Correlation differences between RSFC and Drift Rate vs. RSFC and Start Point for Repeat accumulator. We show a symmetric correlation matrix plot where each square represents the correlation between the row-column DMN resting state functional connectivity and the respective correlation difference between Drift Rate and Start Point.

**Figure 14:**
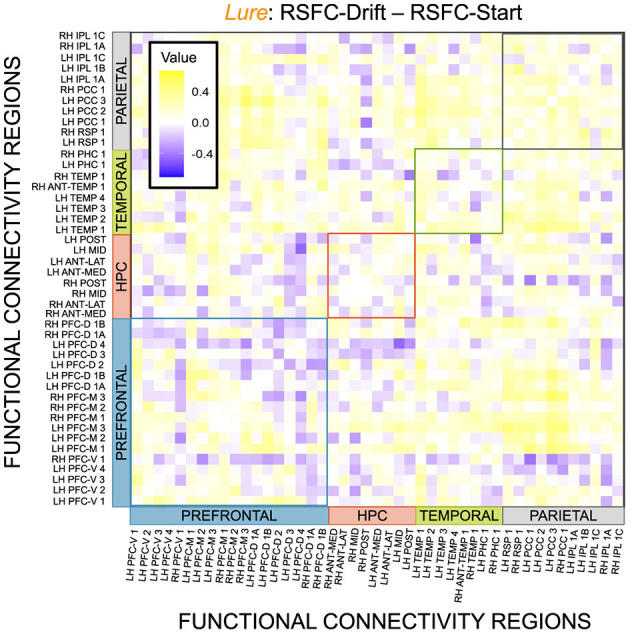
E6: Correlation differences between RSFC and Drift Rate vs. RSFC and Start Point for Lure accumulator. We show a symmetric correlation matrix plot where each square represents the correlation between the row-column DMN resting state functional connectivity and the respective correlation difference between Drift Rate and Start Point.

**Figure 15:**
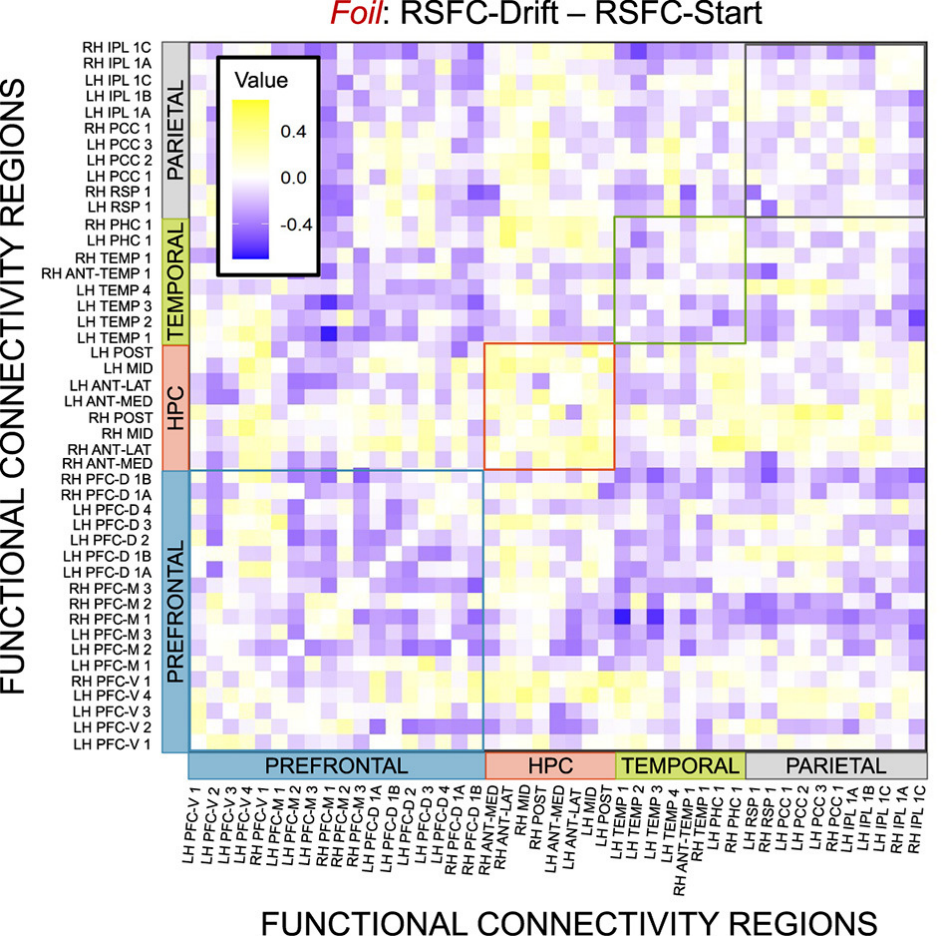
E6: Correlation differences between RSFC and Drift Rate vs. RSFC and Start Point for Foil accumulator. We show a symmetric correlation matrix plot where each square represents the correlation between the row-column DMN resting state functional connectivity and the respective correlation difference between Drift Rate and Start Point.

Given, however, the evidence suggesting pattern separation occurs *throughout* the brain (Amer and Davachi, 2023), we repeated the same analysis examining the relationship between LBA parameters/LDI and resting state functional connectivity in the entirety of the Default Mode Network (DMN). As in the section detailing our behavioral results, we are interested in contrasting the difference in correlations between the respective accumulator drift rates and DMN RSFC with the respective accumulator start points and DMN RSFC. As the drift rates are the parameters that represent signal strength in the MST, we may expect that there will be stronger correlations between the drift rates and DMN RSFC than the start points and DMN RSFC in regions that may support mnemonic discrimination and/or pattern separation. To formally compare dependent (i.e., paired) overlapping (i.e., both relating to RSFC) correlations, we use the R package cocor (Diedenhofen and Musch, 2015) and report results of significance based on the Pearson and Filon's *z* statistic. We show the unthresholded correlation differences between RSFC-Drift and RSFC-Start Point for the Repeat (Figure 13), Lure (Figure 14), and Foil (Figure 15) accumulators.

We then thresholded these correlations using the False Discovery Rate (FDR) via the R package fdrtools (Strimmer, 2008). Across all the accumulators, however, we do not find any correlations where the RSFC-Drift is significantly different from the RSFC-Start Point correlation.

**Figure 16:**
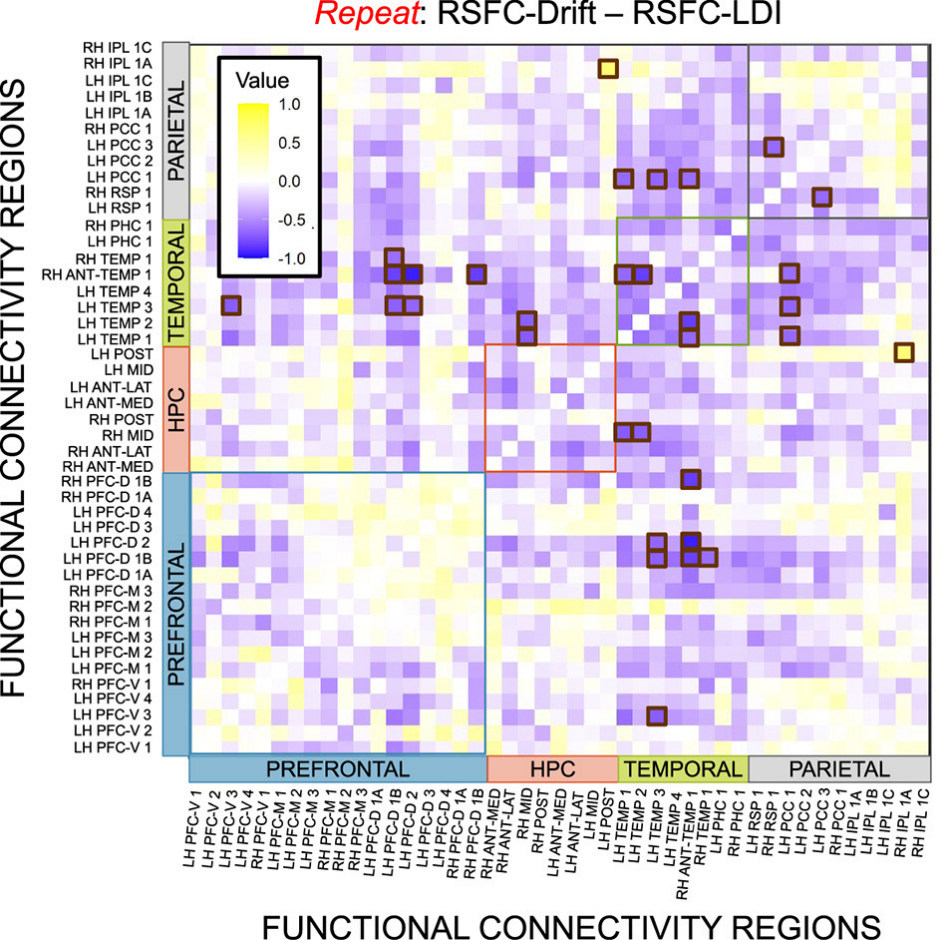
E6: Correlation differences between RSFC and Drift Rate (Repeat Accumulator) vs. RSFC and LDI. We show a symmetric correlation matrix plot where each square represents the correlation between the row-column resting state functional connectivity and the respective correlation difference between Drift Rate and Start Point. Brown squares highlight regions of connectivity where the correlation differences are statistically significant (*p* < 0.05).

We performed a final RSFC correlation difference analysis where we compared RSFC-LDI correlations with RSFC-Drift and RSFC-Start Point (Figure 16). Here we found 16 connectivity regions where the correlation differences survived the statistical signficance threshold via FDR for the Repeat accumulator only. All but one of these regions showed that the RSFC-Drift correlation was more negative than the RSFC-LDI correlation. Of these regions, the most frequently occurring brain region was the Anterior Temporal (Right Hemisphere, RH ANT-TEMP 1, in Figure 16). Specifically, we found RH ANT-TEMP connectivity with 6 (dorsal prefrontal, temporal, and parietal) regions were more negatively correlated with drift than LDI. The only region where RSFC-Drift is more positively correlated than RSFC-LDI is the Posterior Hippocampus (Left Hemisphere) and the Inferior Parietal Lobule (IPL, Right Hemisphere).

## 4 Discussion

In this paper, we have introduced a way to model choice and response time in the Mnemonic Similarity Task. We adapt a version of the Linear Ballistic Accumulator to model each response distinctly: Repeat, Lure, and Foil. A primary contribution of this work is to introduce psychologically interpretable parameters, allowing us to separate signal strength (i.e., drift rate) from other processes (e.g., response bias).

We also demonstrate that the LBA parameters relate systematically with the standard choice-based measure, the LDI. Specifically, that the LDI correlates with *both* signal strength and response bias: it is capturing facets of recognition memory, response tendencies, and other behaviors that evolve over the course of an experiment. The drift rate is a measure of signal strength, or deliberation. In this paper we interpret it as the memory-dependent signal present in mnenomic discrimination. We can further speculate that the decision-related signal captured by the LDI could provide a mechanistic link via evidence accumulation between the LDI and pattern separation itself, though further research is necessary to test this claim. Importantly, while we found variability across experiments in which accumulator drift rates and start point upper bounds correlated with the LDI, our secondary analysis quantifying the difference in correlation strengths showed that the LDI may indeed be capturing more “signal” than response bias (Experiments 1 − 4 only).

To further explore what insights our modeling approach could provide, we considered several other datasets. First, we considered two lifespan samples as age is known to correlate with LDI (Stark et al., 2019). In one experiment (E5), we found differential correlations with LDI and its putative decompositions as a function of age group: LDI correlated with the drift rates for the Repeat and Lure accumulators only for the younger adults, while the start points for Repeat and Foil accumulators only correlated with LDI for the older adults. This suggests that the LDI might also be capturing different processes that adults rely on as a function of their chronological age: younger adults may have strong internal signals, and that is why we see strong correlations with LDI and Drift Rate but older adults may rely on more gisty processes, hence the stronger correlations with LDI and Start Point. Another age related differential correlation we found in E5 showed that age positively correlated with Lure drift rate for the younger group and negatively for the older group. The negative correlation is perhaps unsurprising in the older age group as it is consistent with previous results showing how age and LDI are negatively correlated in older adults (Stark et al., 2019), insofar as the LDI and Lure drift rate capturing similar information. As neurobiological substrates weaken with age, we expect even older people to have even poorer signal strength. However, the converse is not true for the younger age group, the youngest member of which is 18. While hippocampal development is more or less complete by that age, the prefrontal cortex is not. This could be a reason why we see the positive correlation with age in the younger age group alone and could provide further evidence suggesting that a more global neurobiological conceptualization of how/where mnemonic discrimination and perhaps even pattern separation take place in the brain is warranted.

We also examined a dataset that allowed us to examine within-subject test-retest reliability. Specifically, subjects took the standard MST twice – with the second session (E7b) taking place with a one week delay. This dataset allowed us to address the critical notion of information consistency: if parameters were similarly valued or ordered across both sessions, then we may have more consistent parameters. Indeed we found that our LBA parameters both preserved rank correlations for several parameters and also showed relatively small differences in magnitude, especially compared to the LDI.

Finally, we conducted an exploratory analysis to consider whether our model parameters correspond in any way with neural activity. Our results in this paper are focused around explaining the variation in an important behavioral measure of mnemonic discrimination and, possibly, pattern separation. The latter is, however, fundamentally a neural process. Therefore we explored whether our LBA model parameters and LDI differentially correlated with resting state functional connectivity. With hippocampal RSFC, we found that, of all parameters we model, the accumulator drift rates correlated the most with hippocampal RSFC. Critically, we found that *only* the drift rates correlate with the same connectivity regions as the LDI: all in the anterior hippocampus. We also found statistically significant differences between the correlations of RSFC and Repeat accumulator drift rate and RSFC and LDI, where the former was more negative than the latter. While the hippocampus has several functions and there may be other brain regions whose functional connectivity may correlate with both the LDI and start point upper bound, we find these results to be an encouraging step toward beginning to address how “process pure” the LDI may be.

Our findings may enhance the application of MST in several ways. First, the use of sequential sampling models can allow researchers to extract trial-by-trial timeseries reflecting putative underlying computations that drive behavior, which should support analysis of more precisely defined functional neuroimaging measures (Long et al., 2016). Secondly, the robust statistical frameworks often used to fit these sorts of models may allow further refinement of the approach, producing even more stable trait-level estimates by, e.g., incorporating informative priors and models of contaminant behavior, and integrating trial-wise neural measures to simultaneously test mechanistic hypotheses and improve model fit to behavior (Turner et al., 2019). Finally, we draw general attention to how response times can provide meaningful information about an individual's memory discrimination – regardless of whether RT is explicitly modeled and perhaps especially when considering vulnerable or clinical populations.

Our work fits in with other recent process models of performance in this task. One such approach used Multinomial Processing Trees to distinguish remembering and discrimination from each other and from guessing (Lee and Stark, 2023). A key advantage of this model is that it leverages previous psychometric calibrations of lure item similarity (Lacy et al., 2011) to support the distinction of discrimination-based processing, as discrimination should get progressively more difficult with higher-similarity items. However, because it is only fit to choice data, that model may not capture degrees of discrimination processing that doesn't result in meaningful differences in choice probabilities. Thus, a useful future direction for the present model is to adjust our specification of drift rate to account for these levels of similarity.

A second approach used a two-choice evidence accumulation model to identify age-related differences in discrimination, rather than response bias, in the two-response version of the task (Chwiesko et al., 2023). This model is more directly analogous to ours, with the key distinction being in the choice of evidence accumulation framework—namely, the drift-diffusion model (DDM) employed there cannot in principle handle more than two responses. However, the non-ballistic evidence accumulation model may have important advantages over the LBA employed here, namely in that the former captures additive effects of noise during the deliberation process that may explain left-skewed response times in the presence of highly noisy mnemonic information—in other words, capturing errors when our framework might instead identify highly variable drift rates. Here, too, the addition of known similarity structure to the model specification should clarify the distinction between response types and the influence of model assumptions.

Taken together, we believe these findings combine to establish the value of modeling process-level contributions to performance in this widely used task. Further work using sequential sampling frameworks can examine whether these process-level components shed further light on the cognitive (Gallo et al., 2004; Bowman and Dennis, 2016) and neural (Kirwan and Stark, 2007; Bowman and Dennis, 2016) underpinnings of lure discrimination, and whether they can contribute to the growing literature using behavioral markers of mnemonic discrimination to predict neurocognitive degeneration (Berron et al., 2019; Maass et al., 2019; Stark et al., 2019; Webb et al., 2020; Trelle et al., 2021; Kim et al., 2023).

## Data availability statement

Some of the datasets are publicly available, others are available by request to the original authors SN: nohsm@uci.edu, CK: kirwan@byu.edu, and CS: cestark@uci.edu.

## Ethics statement

The CS and SN datasets were collected at University of California, Irvine and approved by the UCI IRB. The CW dataset was approved by the UNC Greenboro IRB. The CK dataset was approved by the BYU IRB. The studies were conducted in accordance with the local legislation and institutional requirements. The participants provided their written informed consent to participate in this study.

## Author contributions

NB: Writing – review & editing, Writing – original draft, Methodology, Investigation, Formal analysis, Conceptualization. SN: Writing – review & editing, Resources. CW: Writing – review & editing, Resources, Methodology. BC: Writing – review & editing, Resources, Methodology. CK: Writing – review & editing, Resources. CS: Writing – review & editing, Resources. AB: Writing – review & editing, Writing – original draft, Supervision, Project administration, Funding acquisition, Conceptualization.
